# Corticospinal tract damage in HHH syndrome: a metabolic cause of hereditary spastic paraplegia

**DOI:** 10.1186/s13023-019-1181-7

**Published:** 2019-08-23

**Authors:** Giorgia Olivieri, Stefano Pro, Daria Diodato, Matteo Di Capua, Daniela Longo, Diego Martinelli, Enrico Bertini, Carlo Dionisi-Vici

**Affiliations:** 1 Division of Metabolism, Department of Pediatric Specialties, Bambino Gesù Children’s Hospital, IRCCS, Piazza S. Onofrio 4, 00165 Rome, Italy; 2 Neurophysiology Unit, Department of Neuroscience, Bambino Gesù Children’s Hospital, IRRCS, Rome, Italy; 3 Laboratory of Molecular Medicine, Unit of Muscular and Neurodegenerative Disorders, Bambino Gesù Children’s Hospital, IRCCS, Rome, Italy; 4 Neuroradiology Unit, Imaging Department, Bambino Gesù Children’s Hospital, IRRCS, Rome, Italy

**Keywords:** HHH syndrome, Hereditary spastic paraplegia, Urea cycle defects, Ornithine

## Abstract

**Background:**

Hyperornithinemia–hyperammonemia–homocitrullinuria (HHH) syndrome is a rare disorder of urea cycle characterized by progressive pyramidal and cerebellar dysfunction, whose pathophysiology is not yet fully understood. Here we describe the spectrum of the long fibers involvement in HHH syndrome, attempting a correlation between clinical, electrophysiological and neuro-radiological data.

**Methods:**

Nine HHH patients were longitudinally evaluated by clinical examination, neurophysiological assessment including motor (MEPs), somato-sensory evoked potentials (PESS) and nerve conduction velocity (NCV), brain and spinal cord MRI

**Results:**

All patients had pyramidal dysfunction and 3/9 an overt spastic paraplegia. Mild to moderate cerebellar signs were found in 7/9, intellectual disability in 8/9. At lower limbs, MEPs resulted abnormal in 7/8 patients and PESS in 2/8; peripheral sensory-motor neuropathy was found in 1/9. MRI documented atrophic changes in supra-tentorial brain regions in 6/9 patients, cerebellum in 6/9, spinal cord in 3/7.

**Conclusions:**

A predominant corticospinal dysfunction is evident in HHH syndrome, along with milder cerebellar signs, intellectual disability of variable degree and rare peripheral neuropathy. Phenotypical similarities with other disorders affecting the urea cycle (argininemia and pyrroline-5-carboxylate synthetase deficiency) suggest possible common mechanisms contributing in the maintenance of the corticospinal tract integrity. HHH syndrome phenotype largely overlaps with complex Hereditary Spastic Paraplegias (HSPs), in the list of which it should be included, emphasizing the importance to screen all the unsolved cases of HSPs for metabolic biomarkers.

## Background

Hyperornithinemia–hyperammonemia–homocitrullinuria (HHH) syndrome (OMIM # 238970), caused by mutations in *ORNT1 (SLC25A15)* gene, is a rare autosomal recessive disorder of the urea cycle [1, 2]. The metabolic triad of hyperammonemia, hyperornithinemia and homocitrullinuria establishes the diagnosis. Clinical manifestations are widely heterogeneous, ranging from neonatal life-threatening hyperammonemic crises to milder forms with onset at variable ages. Once the treatment is started, the clinical course is usually stable, but a progressive lower limb spasticity and a cerebellar dysfunction appear in most cases, leading to mild gait disturbance up to loss of deambulation [3–7]. Intellectual disability of variable degree and, more rarely, focal dystonia and myoclonus may also occur [3–6]. Pathophysiology of pyramidal and cerebellar dysfunction remains to be elucidated. To date, neurophysiological and neuroimaging studies have been conducted only in few patients. Somato-sensory evoked potentials (SSEPs) and peripheral nerve conduction velocity (NCV) were occasionally found to be abnormal [6]. Motor evoked potentials (MEPs) have been rarely performed and, despite pyramidal dysfunction is presumed to be progressive, no longitudinal studies have been reported so far [4, 5]. Similarly, available neuroimaging data are mostly based on anecdotal descriptions [5, 8–12]. Herein we describe neurological, neurophysiological and neuroradiological findings, collected over a 15-year follow-up, in 9 HHH patients. We aimed to correlate long motor and sensitive tracts and peripheral nervous system involvement with neuroimaging and neuro-functional outcome data.

## Patients and methods

We report on 9 patients (5 female, 4 male; mean age at last examination 24.4 ± 16.3 years; range 7–52.6), with genetically confirmed HHH syndrome, regularly followed at the Division of Metabolism of the Bambino Gesù Children’s Hospital in Rome. The early course and molecular abnormalities of 8/9 patients have been reported in previous studies [3, 5, 12–15]. Clinical features are summarized in Table 1. Follow-up had a mean duration of 15.9 ± 12.6 years (range 3–34 years) and encompassed clinical, neurological, neurophysiological and neuro-radiological data, retrospectively reviewed and prospectively integrated whenever necessary.

**Table 1:** Main clinical and genetic features in nine patients with HHH syndrome

Pt Gender (age at last evaluation)	Genotype	Onset	Diagnosis	Previous coma	Previous lethargy	Seizures/ myoclonus	Intellectual disabilty	Adaptive functions		Reference
								Daily activity	Employment/ scholarization	
1 male (53 years)	79G > A/79G > A G27R/G27R	2 yrs	41 yrs	+	+	–	borderline	1	+ (1)	[3, 12]
2 male (44 years)	535C > T/535C > T R179X/R179X	12 yrs	26 yrs	–	+	+	mild	2	none (2)	[3, 5]
3 male (35 years)	861insG/861insG S90X/S90X	14 d	2 yrs	+	+	+	severe	3	none (3)	[3, 5, 14]
4 female (34 years)	79G > A/79G > A G27R/G27R	4 d	3 mo	+	–	+	borderline	1	+ (1)	[3, 5, 13, 14]
5 female (34 years)	824G > A/824G > A R275Q/R275Q	3 yrs	7 yrs	–	+	–	moderate	2	none (3)	[3, 5, 15]
6 male (29 years)	IVS5 + 1G > A/IVS5 + 1G/A (exon skipping)	18 yrs	21 yrs	+	+	+	borderline	3	none (3)	[3, 5]
7^a^ female (12 years)	79G > A/823C > G G27R/R275G	1 yr	8 yrs	–	–	–	borderline	1	+ (2)	[3]
8^a^ female (8 years)	79G > A/823C > G G27R/R275G	1 yr	4 yrs (prospective)	–	–	–	borderline	1	+ (2)	[3]
9 female (8 years)	68G > A/86C > G C23Y/P29R	1 yr	5 yrs	–	–	–	no (selective skills impairment)	1	+ (2)	

Legend: ^a^Siblings. For adaptive functions: 1 = autonomous, 2 = assisted; 3 = dependent. *yrs* years, *mo* months, *d* days, + present, – absent

### Neurological assessment

Spastic paraparesis was assessed according to Harding [16], and the severity of the motor phenotype was evaluated by the Spastic Paraplegia Rating Scale (SPRS) [17]. Central and peripheral somato-sensory signs, and cerebellar signs were evaluated as well.

### Neurophysiological assessment

Long fibers tract assessment encompassed MEPs at upper and lower limbs for the corticospinal tract, and SSEPs at lower limbs for the afferent somato-sensory tracts. Neurophysiological assessments were completed by NCV at lower limbs.

MEPs recordings were performed at the *abductor pollicis brevis* (APB) muscle for the upper limbs, and at the *tibialis anterior* (TA) muscle or, in few cases, at the *abductor hallucis* (AH) muscle for the lower limbs. Assessments consisted in the recording of the central motor conduction time (CMCT) at the target muscle, in both conditions of rest and of slight tonic voluntary contraction (facilitation). CMCT values were considered abnormal if > 20 ms or with a side-to-side difference > 2 ms [18]. Normative CMCT values were obtained from literature data for adult patients [19] and from studies performed at our hospital on healthy age-matched subjects for pediatric ones. SSEPs were measured at the *posterior tibial* muscle by the central sensory conduction time (CSCT). Motor NCV was recorded at the *popliteus sciaticus internus* (PSI) or *popliteus sciaticus externus* (PSE) nerves, and sensory NCV at the *sural nerve*. In all the cases, single assessments encompassed side-to side recordings and mean values between right and left side were considered.

### Neuroradiological assessment

MRI examinations were performed on a 1.5 or 3-Tesla MR scanner, and included sagittal T1w, axial T2w, FLAIR and diffusion weighted images. As comprehensive of all the required sequences, three brain MRI assessments performed in other institutions were considered as well. Images have been evaluated for the evidence of supra-tentorial, sub-tentorial, spinal cord involvement, and corpus callosum abnormalities. To assess the severity of atrophy in each area, and its progression over follow-up, a severity score was designed: - (absent/normal), + (mild), ++ (moderate), +++ (severe).

## Results

### Neurological assessment

Table 2 summarizes the neurological findings at follow-up. All patients showed variable signs of pyramidal dysfunction, and an overt spastic paraplegia was detected in 3/9 patients. The remaining ones showed milder symptoms, such as lower limbs hyperreflexia or bilateral clonus. Seven out of 9 patients had mild to moderate cerebellar signs. Notably, the two patients lacking cerebellar symptoms were the youngest in our cohort. At last examination, all patients were able to walk alone for few meters at least, except for patient #3 who was wheelchair-bound since the age of 20 years. Romberg’s test was assessed in 8/9 patients and resulted negative. Peripheral neuropathy was clinically evident only in patient #3. As assessed by SPRS, spasticity severity reached a median score of 17.8/54 (SD ±15.4), and no correlation was found when SPRS scores were matched with age at evaluation (*p* = > 0.05). Eight out of 9 patients had an intellectual disability of variable degree.

**Table 2:** Main neurological, neurophysiological and neuroradiological findings at last follow-up in nine patients with HHH syndrome

Pt (age at last evaluation)	Higher motor ability	Spastic paraplegia	Pyramidal signs	SPRS (max 52)	Cerebellar signs	Somato-sensory signs		Neuropathy signs	Lower limb neurophysiological findings			
						Romberg Discrimination touch Vibration	Pain Termic Protopatic touch		MEPs (CMCT)	SSEPs (CSCT)	NCV	
									rest/ facilitation		motor	sensory
1 (53 years)	run	–	+	9	+	–	–	–	NE/NE	delayed	normal	normal
2 (44 years)	deambulant with assistance	+	+	31	+	–	–	–	delayed/delayed	normal	normal	normal
3 (35 years)	wheelchair bound	+	+	44	+	nd	nd	+	nd	nd	NE	NE
4 (34 years)	deambulant	–	+	13	+	–	–	–	NE/ normal	normal	normal	normal
5 (34 years)	deambulant	–	+	20	+	–	–	–	NE/normal	normal	normal	normal
6 (29 years)	deambulant with assistance	+	+	37	+	–	–	–	NE/delayed	normal	normal	normal
7 (12 years)	run	–	+	4	+	–	–	–	normal/normal	normal	normal	normal
8 (8 years)	run	–	+	2	–	–	–	–	delayed/delayed	normal	normal	normal
9 (8 years)	run	–	+	1	–	–	–	–	NE/delayed	delayed	normal	normal

Legend: *CMCT* Central motor conduction time, *CSCT* Central sensory conduction time, *FU* Follow-up, *MEPs* Motor evoked potentials, *NCV* Nerve conduction velocity, *NE* Not evocable, *nd* not done, *SSEPs* Somato-sensory evoked potentials,* SPRS* Spastic Paraplegia Rating Scale, + present, – absent

Clinical assessment of the somato-sensory system (performed in 8/9 patients) resulted invariable negative. However, beside some easily recognizable neurological signs (e.g. Romberg test), the ones depending on patient’s perception (e.g. discriminative and protopatic touch, sense of vibration, pain, heat) resulted often difficult to assess, because of the cognitive impairment of the patients themselves.

### Neurophysiological assessment

One patient (#3) was excluded due to peripheral neuropathy, and neurophysiological assessment was finally performed in 8/9 patients.

#### Central motor pathway

**Fig. 1:**
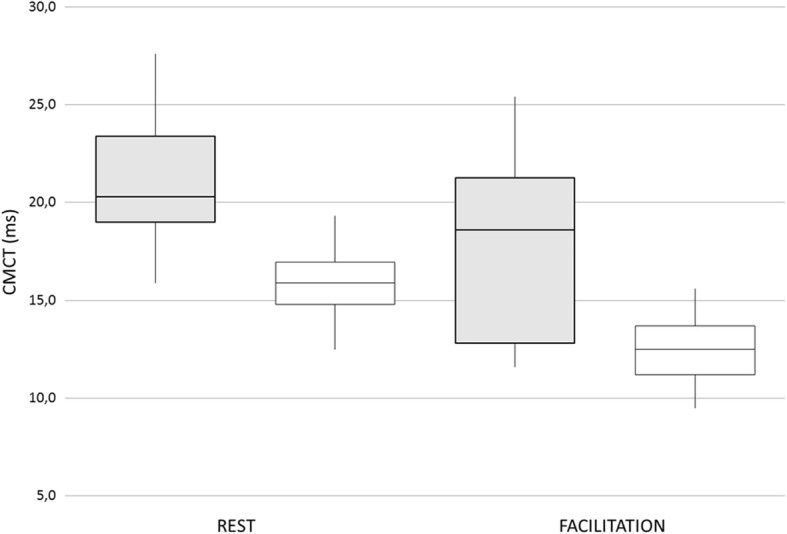
Lower limbs motor evoked potential (MEPs) in eight patients with HHH syndrome. Mean central motor conduction time (CMCT) at rest and during muscle facilitation (*tibialis anterior* muscle) in HHH patients (grey bars) in comparison with healthy controls (white bars). Values are expressed as milliseconds (ms)

Lower limb MEPs were recorded at TA muscle in 7/8 patients and at AH muscle in 1/8 patient (#6). One patient (#4) underwent recordings at both the muscles over its follow-up. Seven out of eight patients were tested at least twice (mean interval between first and last assessment 8.3 ± 6.9 years). Recordings at TA muscles showed mean CMCT 21.2 ± 4.8 ms at relaxed muscle (n.v. 15.9 ± 1.5) and 18.0 ± 4.4 ms at facilitation [n.v.12.5 ± 1.5 [19] for adults; 11.5 ± 1.6 for paediatric patients]. Longitudinal neurophysiological data for each patient are showed in Table 3. Mean TA CMCT values compared to healthy controls are shown in Fig. 1. Recordings at HA muscle showed no evocable response at relaxed muscle and a mean CMCT 21.0 ± 1.7 ms at facilitation [n.v. 16.9 ± 0.9 [20]]. At last follow-up, MEPs resulted pathological (absent or delayed) in 7/8 patients (87.5%) at rest, and in 5/8 (62.5%) at facilitation.

**Table 3:** Longitudinal MEPs values in 9 patients with HHH syndrome

Pt	CMCT (ms) at rest/facilitation				
	I	II	III	IV	V
1	NE/23.5	NE/NE	–	–	–
2	19/18.6	19.5/18.9	20.3/19.45	23.4/20,25	26.35/21.25
3	–	–	–	–	–
4	NE/14.5	NE/15.0	^a^NE/19.2	^a^NE/20.0	–
5	NE/12.1	NE/12.3	–	–	–
6	^a^NE/22.0	^a^NE/22.9	–	–	–
7	16.6/12.5	21.8/12.8	15.9/11.6	–	–
8	27.6/22.8	–	–	–	–
9	NE/25.4	NE/17.45	NE/21.95	–	–

Legend: recordings were performed at *tibialis anterior* muscle (TA), or at *abductor halluces* (AH) muscle^a^. *NE* Not evocable, – not done

Upper limb MEPs were recorded in 3/8 patients, with one patient tested twice at an interval of 5 years. Values resulted within the normal range with a mean CMCT 9.9 ± 1.7 ms [n.v. 7.9 ± 2.1; +3SD =14.2 [21]].

#### Central somato-sensory pathways

All patients but one (#5) were tested at least twice (mean interval between first and last assessment 7.1 ± 6.9 years). Mean CSCT was 19.8 ± 3.9 ms [n.v. 16.4 ± 1.4; +3SD = 20.6 [22, 23]]. Patient # 8 showed a progressively improving trend from infancy to adolescence, reaching borderline/normal values at 9 years. At last follow up, 2/9 patients showed abnormal responses (#1, 9). No correlation between SSEPs values and clinical findings were found.

#### Peripheral nerve conduction

Assessments included 14 PSI, 10 PSE and 16 sural nerve recordings in 8/9 patients. Six patients were tested at least twice (mean interval between first and last evaluation 6.7 ± 9.6 years). Eight out of 9 patients showed normal results [24]. Patient #3 showed progressively worsening NCVs with no evocable responses since the age of 18 years.

### Neuroradiological findings

**Fig. 2:**
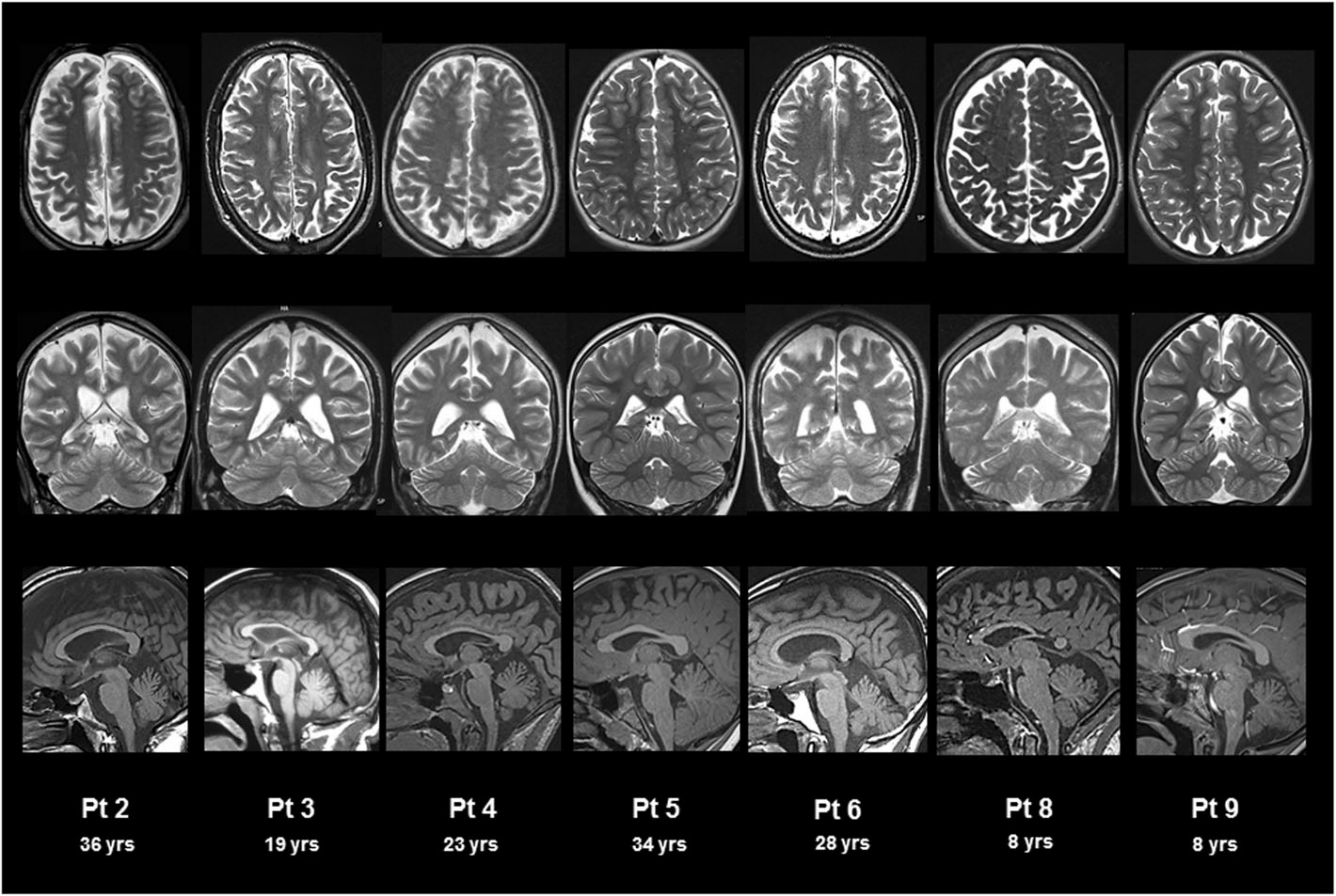
Brain MRI in patients with HHH syndrome. T2 weighted axial (upper panel), coronal (middle panel), and T1 weighted sagittal (lower panel) brain MRI. Atrophy of variable degree is detected in supra-tentorial region (moderate in patients #2, 3, 5 and 6; mild in patient # 4; absent in patients #8, and 9), corpus callosum (moderate in patients #3 and 5; mild in patients # 2, 4 and 6; absent in patients # 8 and 9) and cerebellum (mild in patients #2, 4, 5, 6 and 9, absent in patients # 3, and 8)

Assessments included 14 brain and 7 spinal cord MRI. All patients underwent at least one brain MRI. Brain neuro-radiological findings at follow-up are shown in Fig. 2. Atrophic changes were found in supratentorial and/or subtentorial regions in 7/9 patients. As shown in Table 4, atrophy involved supra-tentorial white matter in 6/9 patients, mild in 2 and moderate in 4. As for sub-tentorial regions, 6/9 patients showed mild atrophic changes of cerebellar vermis. Corpus callosum abnormalities were found in 5/9 patients with mild atrophy in 3 and moderate in 2. Four out of 9 patients (# 2, 4, 5, 6) underwent two or more brain MRI (mean age at baseline 19.4 ± 10.0 years; mean interval between first and last assessment 8.7 ± 2.9 years) with two patients (#2 and 5) showing a worsening trend over years.

**Table 4:** Main neuroradiological findings over follow-up in 9 patients with HHH syndrome

Pt	Age at MRI assessment (yrs)	Brain atrophy			Spinal cord atrophy
		Supratentorial	Corpus callosum	Subtentorial	
1	40	+	–	+	n.d.
2	27	+	–	–	n.d.
	34	++	+	–	n.d.
	36	++	+	+	++
3	19	++	++	–	+++
4	18	+	+	+	n.d.
	23	+	+	+	+
5	25	++	+	–	n.d.
	34	++	++	+	–
6	28	++	+	+	n.d
7	12	–	–	–	–
8	8	–	–	–	–
9	8	–	–	+	–

Legend: +, mild; ++, moderate; +++, severe; absent, −. *n.d* not done

**Fig. 3:**
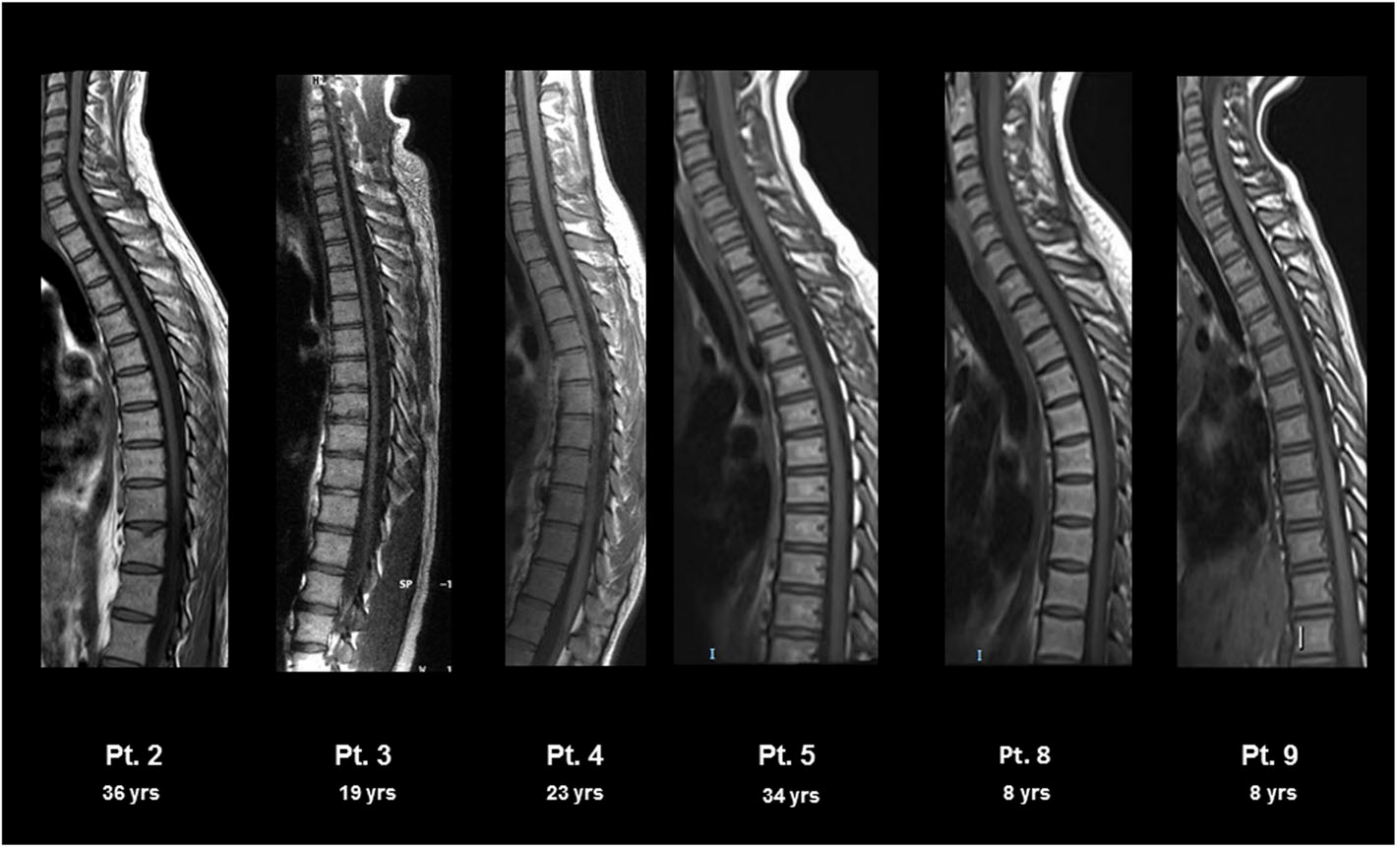
Spinal cord MRI in patients with HHH syndrome. T1 weighted spinal cord MRI showed atrophy of variable degree (severe in patients #3, moderate in patient #2, mild in patient #4, and absent in patients #5, 8 and 9)

Neuroradiological findings at spinal cord are shown in Fig. 3. Spinal cord atrophy was detected in 3/7 patients (Table 4). In one patient (#3), the neuroradiological features were consistent with the particularly severe clinical and neurophysiological outcome.

## Discussion

This study reports on the longitudinal description of neurological, neurophysiological and neuroradiological patterns in HHH syndrome. In our cohort, pyramidal signs were invariably present, with an overt picture of spastic paraplegia detectable in one third of the cases. Other clinical features encompassed a cognitive impairment of variable degree, a frequent cerebellar dysfunction, and more rarely a secondary peripheral neuropathy, which enlarges the spectrum of the phenotypic disease severity. Characteristically, clinical signs of central sensory dysfunction were never observed. Neurophysiological data confirmed a prominent involvement of the primary motor descending pathway compared to the long fibers sensory tracts, and a less frequent involvement of peripheral nerve. Neuroradiological studies, despite not completed by diffusion tensor imaging techniques, confirmed the prominent affection of the regions anatomically corresponding to pyramidal tract (frontal subcortical white matter and, in the most severe cases, cortical gray matter as well). With regard to the final clinical outcome, our findings show heterogeneous profiles, which are apparently independent from the age at evaluation, from the age of treatment initiation, as well as from the length of the disease course, which is particularly difficult to assess in patients who never experienced overt hyperammonemia, which correspond to one third of our cohort.

The association of lower limbs spasticity with mild cerebellar signs, cognitive impairment, and possible neuropathy indicates that HHH syndrome should be listed among the hereditary spastic paraplegias (HSPs). In particular, given its composite phenotypic picture, HHH syndrome fulfills the diagnostic criteria of complex HSPs [25].

Nowadays, HSPs encompass more than 80 loci and over 60 genes [26, 27], nevertheless, many conditions remain undiagnosed, suggesting that new genetic determinants still remains to be identified.

With regard to neurophysiological findings in HHH syndrome, our data are consistent with the clinical picture, highlighting the predominant involvement of the descending motor tracts compared to the ascending sensory ones. MEPs recorded at lower limbs showed delayed or not evocable response in about 90% of our patients. This occurred in both adults and children, showing a progressive worsening trend over time. A slowing-down, up to the loss, of an evocable response of CMCT was mostly detectable at rest at first evaluation, but it progressively involved the response also at muscle contraction at follow-up. This is in line with the neurophysiological response to a progressive neuronal degeneration in which a pathological response at rest can be hided or mitigated in facilitation conditions, when higher threshold and faster pyramidal neurons are recruited [28, 29]. What follows is that the SPRS scale, which scores spastic paraplegia according to patient’s age and disease duration, is not an optimal tool to quantify the severity of pyramidal dysfunction in HHH syndrome.

Contrarily to MEPs, SSEPs resulted unaltered in the most of our patients. Accordingly, clinical signs of sensory defects were never recorded, not even in the two patients who showed mildly delayed SSEPs values.

Neuroradiological data also confirm a slow progression of the disease. Atrophy primarily affects the subcortical white matter and, in the most severe cases, mildly involving cortical gray matter as well, whilst cerebellum is less affected. These findings are in line with literature reports on brain lesions observed in urea cycle disorders, which selectively and primarily affects the deep white matter [30]. Since the disease course in HHH syndrome is usually stable, with low risk of hyperammonemia once diet and pharmacological therapy are started, brain lesions are milder if compared to other urea cycle disorders, presenting a more severe long-term course [31, 32]. Therefore, brain abnormalities detected in HHH syndrome are likely to be not only dependent on the severity and duration of hyperammonemia alone. In fact, the 4/9 patients in our cohort who experienced hyperammonemic coma showed heterogeneous MRI findings: #3 and #6 had the most severe clinical and neuroradiological outcome, while #1 and #4 had mild lesions, comparable to those of patients who never experienced severe hyperammonemia.

Concerning spinal cord, MRI changes suggest a correlation with the clinical outcome, with the most severe atrophic degeneration observed in patients with the higher SPRS score (patient #3 who is wheelchair bound, and patient #2 who hardly maintains autonomous gait, only for a few steps). Although spinal cord atrophy progression rate cannot be determined since patients underwent a single MRI study, the ongoing trend of neurophysiological assessments suggest a similar evolution.

**Fig. 4:**
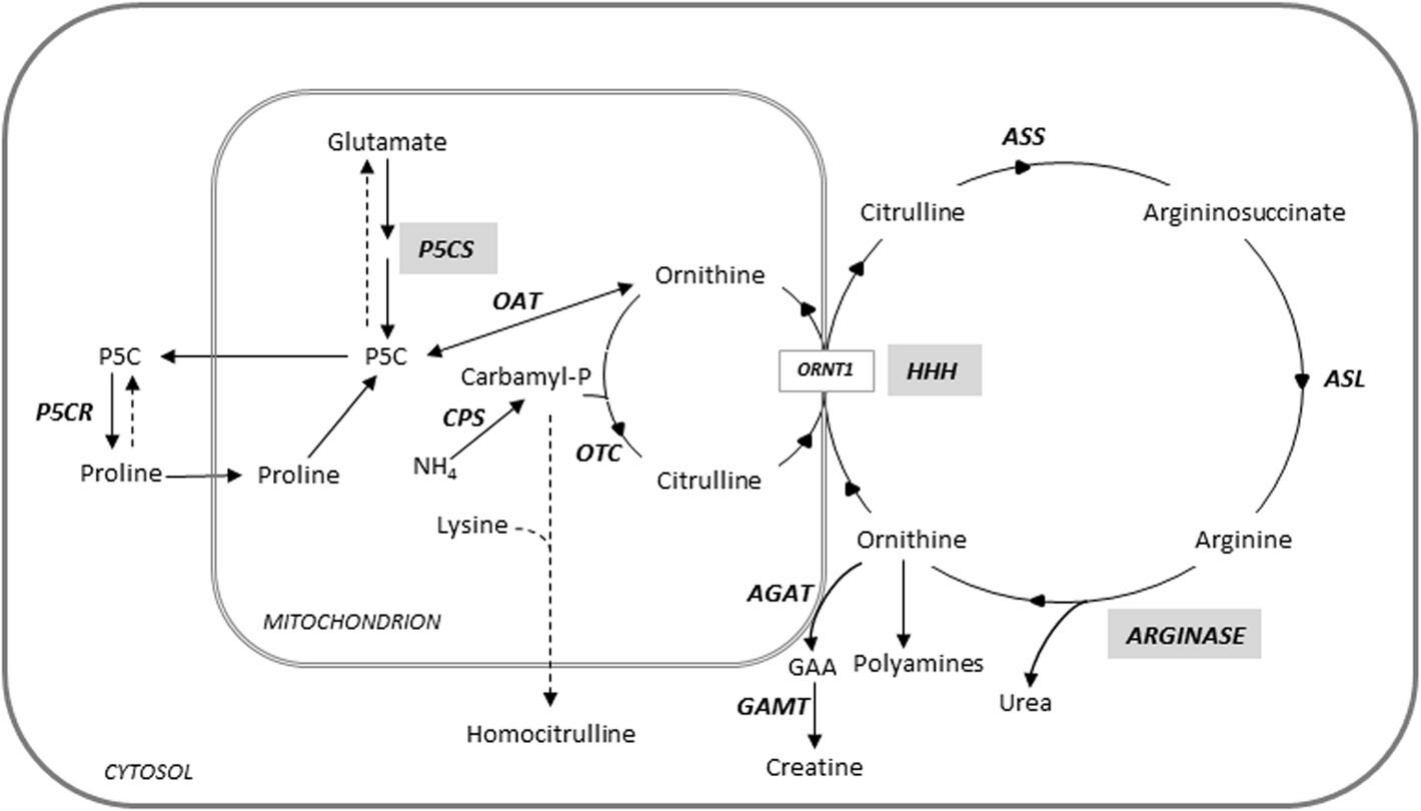
Urea cycle and related pathways. The illustration shows the biochemical pathways connecting HHH syndrome, argininemia and pyrroline-5-carboxylate synthetase deficiency (gray boxes), the three disorders of aminoacid metabolism related to the distal part of the urea cycle sharing phenotypic similarities. AGAT, l-arginine:glycine amidinotransferase; ASL, argininosuccinate lyase; ASS, argininosuccinate synthetase; CPS, carbamyl-phosphate synthetase; GAA, guanidinoacetate; GAMT, guanidinoacetate N-methyltransferase; OAT, ornithine aminotransferase; ODC, ornithine decarboxylase; ORNT1, ornithine/citrulline antiporter; OTC, ornithine transcarbamylase; P5C, pyrroline-5-carboxylate; P5CR, pyrroline-5-carboxylate reductase; P5CS, pyrroline-5-carboxylate synthase

Clinical, neurophysiological and neuroradiological findings in HHH syndrome resemble those observed in argininemia and pyrroline-5-carboxylate synthetase (P5CS) deficiency [33–36], two other disorders of aminoacid metabolism connected to the distal part of the urea cycle (Fig. 4).

It is noteworthy that, as in HHH syndrome, these diseases are associated with spastic paraplegia, variable cognitive impairment and possible cerebellar signs, then suggesting possible common mechanisms connecting urea cycle-related pathways with the maintenance of the cortico-spinal tract integrity [37]. Possible shared mechanisms include abnormalities of arginine, creatine, polyamines, and proline metabolism, and dysregulation of the autophagy machinery, this last representing a known cause of some HSPs [38].

## Conclusions

Our study highlights the progressive involvement of the corticospinal tract in HHH syndrome. With regard to neurophysiological data, corticospinal system dysfunction resulted clearly predominant if compared to central sensory system and to peripheral nervous system. MEPs showed a pathological pattern with a progressive worsening trend with age. Although the cause of the selective involvement of cortical spinal tract in HHH syndrome remains to be elucidated, the similarities with argininemia and P5CS deficiency, suggest possible common pathophysiological mechanisms.

The presence of pyramidal signs/lower limb spasticity associated with cerebellar signs and cognitive impairment, highlights the importance to list HHH syndrome among the complex form of HSPs.

## Data Availability

All data which have been generated and analyzed during this study are included in the article.

## Abbreviations

AH: *Abductor halluces*
APB: *Abductor pollicis brevis*
CMCT: Central motor conduction time
HHH: Hyperornithinemia–hyperammonemia–homocitrullinuria
MEPs: Motor evoked potentials
NCV: Nerve conduction velocity
P5CS: Pyrroline-5-carboxylate synthetase
PSE: *Popliteus sciaticus externus*
PSI: *Popliteus sciaticus internus*
SPRS: Spastic paraplegia rating scale
SSEPs: Somato-sensory evoked potentials
TA: *Tibialis anterior*
